# Ultra-trace level detection of arsenic and selenium using a commercially available hydride generator with atomic-absorption detection

**DOI:** 10.1155/S1463924683000474

**Published:** 1983

**Authors:** R. W. Ward, P. B. Stockwell

**Affiliations:** 1 RF Plasma Products Inc. 22 Olney Avenue Cherry Hill New Jersey 08003 USA; 2 P. S. Analytical Ltd 2 Eagles Drive Tatsfield, Westerham Kent TN16 2PB UK


Journal of Automatic Chemsitry, Volume 5, Number 4 (October-December 1983), pages 193-196

### Ultra-trace level detection of arsenic and selenium using a commercially available hydride generator with atomic-absorption detection

**R. W. Ward**

*RF Plasma Products, Inc., 22 Olney Avenue, Cherry Hill, New Jersey 08003, USA*

**and P. B. Stockwell**

*P. S. Analytical Ltd, 2 Eagles Drive, Tatsfield, Westerham, Kent TN16 2PB, UK*

### Introduction

This paper describes the application of a simple, well-designed automated system for the generation of hydrides from arsenic, selenium and similar elements, prior to their analysis by atomic absorption or inductively coupled plasma emission. The design concepts involved are based on continuous-flow principles and the sample measurements are taken directly above a base-line established by continuously pumping blank solution. Flow characteristics of the gas/liquid separator are designed to ensure that the hydrides formed are swiftly transferred into the atom cell or ICP torch, and that they have a low residence time or memory effect. Coupled together, these features allow sample analysis times to be reduced to an acceptably low level. The ease of use, reliability of the unit and the performances achieved, using either staff unfamiliar with the equipment or untrained, are put forward as prime examples of automating simple chemical sample-preparation techniques to achieve analytical goals. The basic requirement always being that if a simple system is designed, it will be easier to use and maintain.

The use of hydride generation to assist in the analysis of many metalloidal elements has proved extremely popular, as evidenced by the wealth of published literature [1–3]. Godden and Thomerson [1] recently published an extensive review on this topic, which provides an excellent introduction to the area. The techniques have been developed primarily for arsenic and selenium, however, many more elements are achieving attention, for example bismuth, tellurium and geranium. Some years ago, attention was focused on mercury and it is now possible to analyse this element using some of the equipment developed. The toxicity of very low levels of these elements necessitates trace level analysis. The low wavelength of their resonance lines, coupled with the low nebulization efficiency of atomic absorption techniques, has led to the popularity of hydride generation methods since 100% transport efficiency of the element into the atom cell is possible. This factor alone significantly increases the detection capabilities for analysis. Continuous-flow hydride generation methods have also been successfully coupled with plasma emission techniques [3 and 4].

### Chemistry and atomic cells

The basic requirement for the generation of covalent hydrides is a supply of hydride ions. Various reduction cells have been used [1–28], including mixtures of potassium iodide/zinc powder/tin [11], chloride [5–7], or titanium (III) chloride/magnesium powder [8], or aluminium slurry/acid [9]. Schmidt and Royer [10] provided a benchmark in the development of hydride generation for analytical purposes by proposing the use of sodium tetrahydroborate (III) as reductant. This has since proved an extremely popular reductant [1, 3, 4 and 11–13].

The major advantages of using sodium tetrahydroborate (III) as reductant are its ability to generate hydrides from As, Bi, Ge, Pb, Sn, and Te, its higher conversion efficiency, lower blank levels and the rapidity of the reaction. This latter point has negated the use of collection devices [14 and 15], prior to introduction into the atom cell, which in turn decreases analysis time. The instability of the sodium tetrahydroborate (III) solution can be overcome by making the solution 0·1 mol $dm^{-3}$ in sodium hydroxide [16], after filtration through a membrane filter (0·45 $\mu$m) the solution is usable for two to three days. The concentration of this solution has been found to be optimal at around the 1% m/v level by several authors [4 and 16] for various hydride-forming elements with a flow rate of approximately half that of the sample stream.

The relationship between acid concentration and sensitivity was studied by Thompson *et al.* [4] for a variety of acids. The acid of choice was hydrochloric and an optimum concentration of 5–6 mol $dm^{-3}$ existed for both arsenic and selenium. This was later confirmed by Ebdon *et al.* [16], who also demonstrated that arsenic and selenium solutions could be stabilized for several days, at the 0·1 $\mu g\,ml^{-1}$ level, by the addition of sodium iodide (1% m/v) and sodium bromide (1% m/v) respectively.

The relative opaqueness of the air/acetylene flame at the arsenic and selenium wavelengths [23] has led to various atomization schemes proving popular. The greater transparency of the argon or nitrogen/hydrogen diffusion flames has made this a popular atom cell [14, 16 and 23–24]. The generation of gaseous hydrides and the resultant separation of analyte element from possible interferant obviates the problems of compound formation normally associated with this flame. The use of silica tubes either flame [13] or electrothermally heated [19 and 25–27] have the advantage of giving longer atomic residence times and hence improved sensitivity over most flame systems. The generation of hydrogen as a byproduct of the hydride reaction can cause problems if background correction is not used. Ebdon *et al.* [16] demonstrated that a miniature argon/hydrogen diffusion flame can yield detection limits as good as those obtained with the tube furnaces; however, the authors had to choose the silica tube furnace as the atomization cell because this system is not commercially available. The tube was flame heated using a system shown to be simple, robust and readily demountable [28 and 29].

### Continuous or batch operation

The vast majority of publications use batch devices which have been automated to a greater or lesser extent. The device described by Renoe [30] presents a state-of-the-art batch system. However, these systems are subject to one major disadvantage—the hydrogen and hydride formed in the reaction chamber are forced into the flame atom cell simultaneously and so disturb the equilibrium conditions (the measured signal being the sum of two disturbances). In many batch systems the apparatus has to be disassembled after one analysis.

In contrast, the continuous approach relies on the continuous generation of hydrogen and a steady flow into the analysis device. Despite some objections, both AA analysis and ICP systems can accomodate a reasonable level of hydrogen input without affecting the system. Goulden and Brooksbank [9] described an analytical system based on Technicon Autoanalyser technology; however, this pumped an aluminium slurry and sulphuric acid streams—the latter to dry the hydride prior to entry into an atom cell. Stockwell [31] discussed the inherent disadvantages of the Goulden system and outlined the advantages of the one developed by Dennis and Porter [33]. This system, whilst based on good automation design, was designed to overcome a particular analytical problem: selenium in waste water, and has one major disadvantage in that it is not an inherently simple instrument.

Automating the sodium borohydride system based on continuous-flow principles represents the most reliable approach to commercialization.

Flow-injection analysis systems have been described in the literature but do not offer any degree of simplification. The reliability and ease of use of the system used in this work are put forward as a valid argument for accepting the continuous-flow approach as the method of choice.

### Experimental

### *Reagents*

Unless otherwise stated, all reagents were of analytical grade. Sodium tetrahydroborate (III) (lg, 98%, Aldrich Chemical Co.) was dissolved in sodium hydroxide solution (1·01, 0·1 mol $dm^{-3}$) and filtered prior to use.

The diluent for the arsenic and selenium standards were stabilized with sodium iodide (1% m/v) and sodium bromide (1% m/u) respectively.

### *Flow rates*

The sodium tetrahydroborate (III) solution was pumped at 4·7 ml/min and the acid blank/standard solution at 9·4 ml/min with a constant-speed peristaltic pump. The different flow rates were achieved by using 0·5 and 0·8 mm i.d. silicone tubing respectively.

### *Hydride generator*

Figure 1 shows a detailed picture of the commercial hydride generator (Plasma-Therm Ltd, Kangley Bridge Road, London SE26 5AR) used. The reagents, hydrochloric acid and sodium tetrahydroborate (III), were pumped from plastic containers (2·5 1), sited on the left-hand side of the unit, through silicone tubing by a constant-speed peristaltic pump. A separate channel exists for the introduction of sample or standard solutions into the system.

The flow pattern of the system is represented schematically in figure 2. The reducing agent always flows to the Kel-F mixing 'T' whilst the blank acid and sample solutions flow to a six-port, pneumatically actuated Teflon valve.

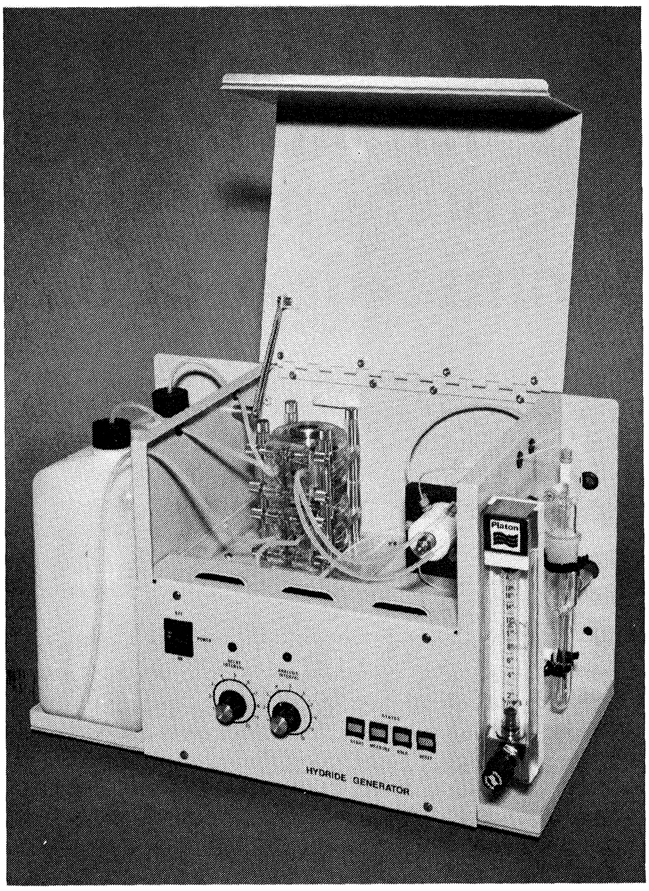

*Figure 1. The new unit.*

The unit operates two sampling cycles, a time period (Tl, 0-200s) during which the blank acid is passed to the 'T' piece and the sample solution goes directly to drain. The second period (T2, 0-300s) occurs immediately after T1, and during this time the sample solution goes to the 'T' piece and the blank acid to the drain. This switch in flows occurs at the end of T1, and is achieved by the pneumatically actuated six-port valve. The use of a six-, rather than four-, port valve enables a smooth transition between acid blank and sample flows.

The reduction occurs at the 'T' piece and is complete by the time the flows reach the glass gas/liquid separator. At this point, the gaseous products, either hydrogen or hydrogen and hydrides, are separated from the liquid products. The latter flowing via a 'U' tube to a free-running drain and the former being purged, by nitrogen, into the atom cell.

The type of signal obtained is shown in figure 3. Between A and B, during time period T1, the blank acid is passing to the mixing 'T' and the hydrogen generated enables the blank level to be monitored. At B, T1 ends and the pneumatic valve switches the sample stream to the 'T' piece. The sample solution flows to the 'T' piece during the T2 period, BD, allowing the analyte signal to be monitored above the blank acid level. It can be seen that it takes the signal a finite time, Tr, to rise to its maximum level. This time is a function of the dead space between the switching valve and the atom cell.

Obviously, any analytical measurements taken between B and C would be meaningless; so once the Tr has been characterized for the system it may be set using a variable pre-set timer on the unit. With this time set, the operator is given a visual indication on the front-control panel as to when the signal has reached maximum and so when to commence analytical measurements. At the end of T2, point D, the blank acid stream is switched to the mixing 'T'. Again, it takes a finite time, Tm, D

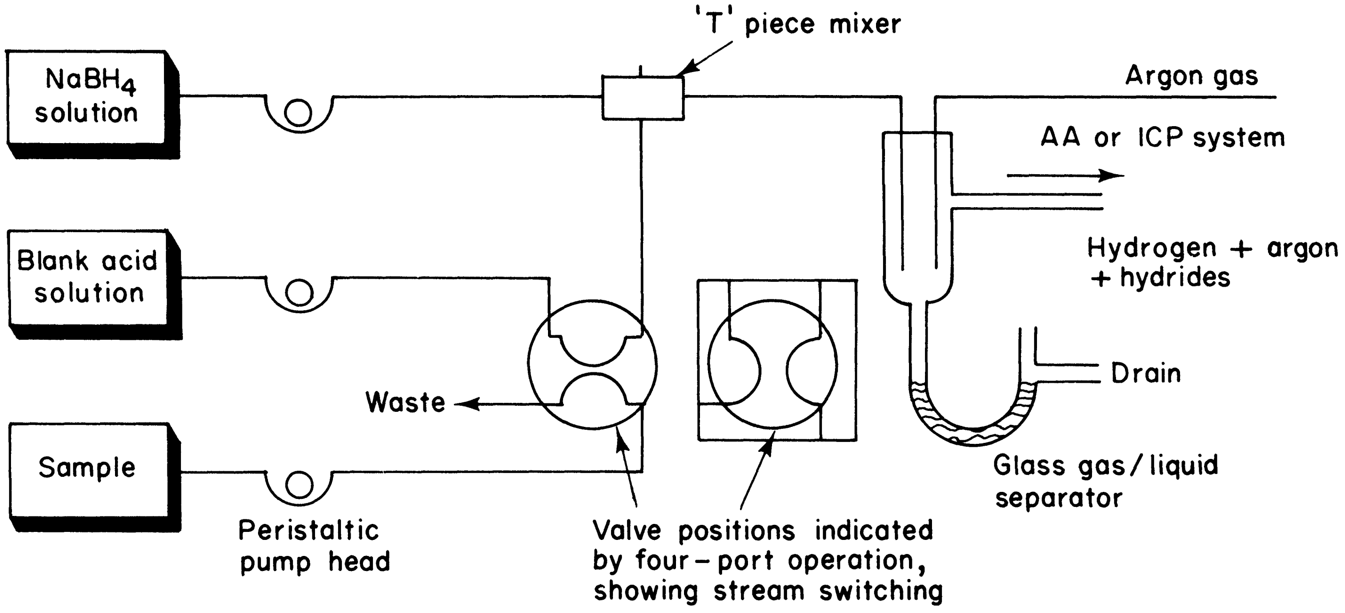

*Figure 2. Schematic flow diagram for continuous-flow hydride generator.*

to E, for the signal to decay back to the blank level. This time is also related to the dead volume of the system. It would be unwise to start another analysis cycle before the blank level has been achieved. Therefore, once Tm has been characterized, it is set on a variable timer, on the back of the unit, and a visual indicator on the control panel informs the operator when to start a new cycle. The setting of Tr and Tm is, if a constant purge-gas flow rate is maintained, a once-only operation. After the time periods T1 and T2, Tr and Tm have been set, the unit only requires the start button to be depressed to go through the cycle A–E.

The unit possesses one additional feature which is extremely useful in the initial optimization of the analysis; it may be held indefinitely in either T1 or T2 modes. Holding the time cycle in T2 enabled variation of the purge-gas flow rate until the optimum flow was achieved.

### *Detection system*

An SP9 atomic absorption system (Pye Unicam Ltd, York St., Cambridge, UK) was used with suitable burner modifications [29] to support the silica 'T' tube. The air and acetylene flows were set so as to provide a stoichiometric flame. The signal from the spectrometer was displayed on both a chart recorder (PM9221, Pye Unicam Ltd) and a reporting integrator (3390A, Hewlett-Packard, Pennsylvania, USA).

The operating conditions for both the hydride generator and spectrometer are given in table 1.

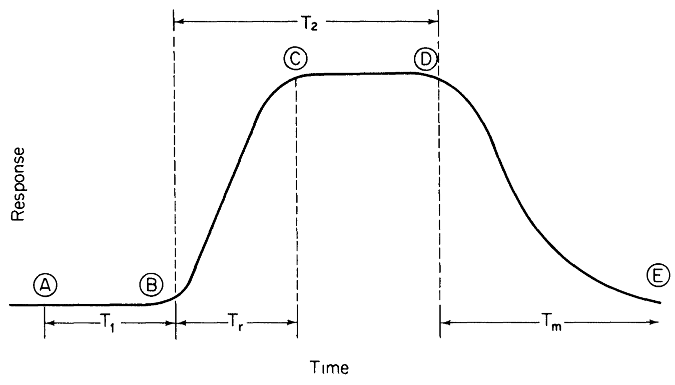

*Figure 3. Typical signal produced from the hydride unit.*

### Results and discussion

The efficiency of operation of the hydride generator may be judged by three criteria; namely throughput, detection levels obtained, and precision of results.

The short time the system takes to reach the maximum signal, Tr, from the onset of T2 and the rapid decay back to the background level, Tm, enable large sample throughputs (see figure 4). The short rise time also reduces the amount of solution required for an analysis; this is important because continuous-flow systems are notoriously profligate with sample solution. Figure 3 shows the peak obtained when initially setting up the system and shows that Tr = 12·5 s and Tm = 14 s. Thus the Tr and Tm timers on the unit were set at 15 and 20 s respectively. This resulted in the peak maximum and resumption of blank levels had been achieved before the visual indicators informed the operator.

*Table 1. Operating conditions.*

| *Hydride generator* | | |
|---|---|---|
| Sodium tetrahydroborate flow rate (ml/min) | | 4·7 |
| Blank acid flow rate (ml/min) | | 9·4 |
| Sample flow rate (ml/min) | | 9·4 |
| Nitrogen purge-gas flow rate (ml/min) | | 550 |
| T1 (s) | | 15 |
| T2 (s) | | 50 |
| Tr (s) | | 15 |
| Tm (s) | | 20 |
| *Spectrometer* | | |
| | Arsenic | Selenium |
| Wavelength (nm) | 193·7 | 196·0 |
| Bandpass (nm) | 1·0 | 1·0 |
| Time constant(s) | 0·1 | 0·1 |
| Mode | Absorption | Absorption |
| Background corrector | Low | Low |
| Lamp current (mA) | 4·0 | 4·2 |
| Air (1/min) | 5·0 | 5·0 |
| Acetylene (1/min) | 1·2 | 1·2 |
| Silica tube-burner | | |
| Separation (mm) | 10 | 10 |

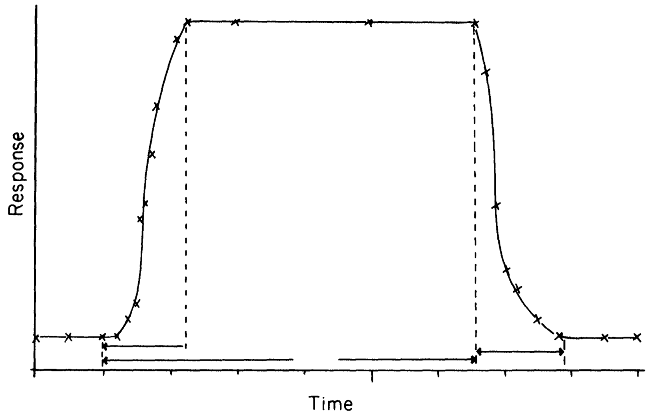

*Figure 4. Initial signal obtained with the system.*

The difference in signals obtained by outputting the data on both a chart recorder and reporting integrator are shown in figure 5. The latter device is normally used in recording chromatographic peaks, thus by setting the peak with equal to T2 the integrator acts as a filter for the noise on the signal. The integrator also yielded both peak height and peak area data. No significant increase in precision was gained by using peak area measurements thus peak height was used.

The precision of the technique was 2·3% rsd and 4·2% rsd at the 20 and 0·5 ng/ml levels respectively. This is significantly better than the precision attainable with manual injection methods.

The detection limits obtained using this system were 0·04 and 0·07 ng/ml for arsenic and selenium respectively which are competitive with those obtained by other workers in the field. The linear ranges obtained were short, up to 80 and 120 ng/ml for arsenic and selenium respectively, and, as for any absorption technique, are the major disadvantage of the method.

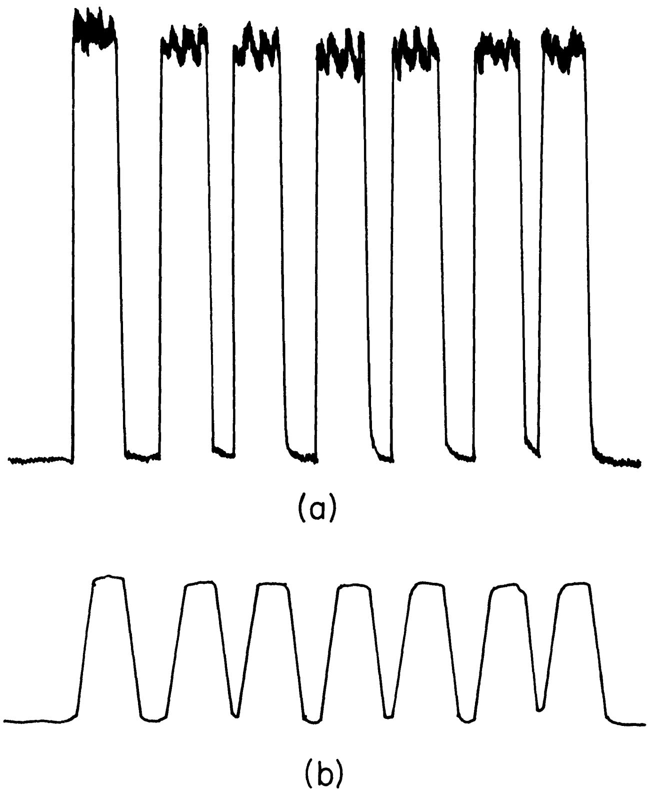

*Figure 5. Seven replicates of sample analysed. (a) Using the chart recorder; (b) using the recording integrator.*

### Conclusion

On the basis of the criteria outlined above, the Plasma-Therm hydride generator is a highly efficient unit. It allows rapid, precise, and sensitive analysis; in addition, it is simple to use. The continuous-flow operation and single-button operation requires a lower level of operator expertise and thus can prove cost-effective in a laboratory situation. The unit may accept and relay signals to an external computer to facilitate not only computer operational control but also computer data handling.

The efficiency and simplicity of operation coupled with the unit's robustness make it ideal for routine usage for wide variety of samples. The unit has been used [33] for low level analysis of mercury by the cold-vapour technique using the same acid and reductant concentrations as outlined above.

### Acknowledgement

The authors would like to thank Dr L. C. Ebdon and the Department of Environmental Sciences at Plymouth Polytechnic for time on their SP9 spectrometer.

### References

1. Godden, R. G. and Thomerson, D. R., *Analyst*, **105** (1980), 1137.

2. Gunn, A. M., *WRC Technical Report, TR-169* (1981), 75.

3. Robbins, W. B. and Caruso, J. A., *Analytical Chemistry*, **51** (1979), 889A.

4. Thompson, M., Pahavanpour, B., Walton, S. J. and Kirkbright, G. F., *Analyst*, **103** (1978), 568.

5. Maruta, T. and Sudoh, G., *Analytica Chimica Acta*, **77** (1973), 37.

6. Korenaga, T., *Analyst*, **106** (1981), 40.

7. Walker, H. H., Runnels, J. H. and Merryfield, R., *Analytical Chemistry*, **48** (1976), 2056.

8. Pollock, E. N. and West, S. J., *Atomic Absorption Newsletter*, **12** (1973), 6.

9. Goulden, P. D. and Brooksbank, P., *Analytical Chemistry*, **46** (1974), 1431.

10. Schmidt, F. J. and Royer, J. L., *Analytical Letters*, **6** (1973), 17.

11. Hon, P. K., Lau, O. W., Cheung, W. C. and Wong, M. C., *Analytica Chimica Acta*, **115** (1980), 355.

12. Evans, W. H., Jackson, F. J. and Dellar, D., *Analyst*, **104** (1979), 16.

13. Thompson, K. C. and Thomerson, D. R., *Analyst*, **99** (1974), 595.

14. Manning, D. C., *Atomic Absorption Newsletter*, **10** (1971), 123.

15. Freeman, H. E. and Uthe, J. F., *Atomic Absorption Newsletter*, **13** (1974), 75.

16. Ebdon, L., Wilkinson, J. R. and Jackson, K. W., *Analytica Chimica Acta*, **136** (1982), 191.

17. Schmidt, F. J., Royer, J. L. and Muir, S. M., *Analytical Letters*, **8** (1975), 123.

18. Vijan, P. N. and Wood, G. R., *Talanta*, **23** (1976), 89.

19. Chu, R. C., Barron, G. P. and Baumgarner, P. A. W., *Analytical Chemistry*, **44** (1972), 1476.

20. Arbab-Zavar, M. H. and Howard, A. G., *Analyst*, **105** (1980), 744.

21. Agemien, H. and Thomson, R., *Analyst*, **105** (1980), 902.

22. Agemien, H. and Bedek, E., *Analytical Chemistry*, **119** (1980), 323.

23. Kahn, H. L. and Schallis, J. E., *Atomic Absorption Newsletter*, **7** (1968), 5.

24. Guimont, J., Pichette, M. and Rheaume, N., *Atomic Absorption Newsletter*, **16** (1977), 53.

25. Wauehope, R. D., *Atomic Absorption Newsletter*, **15** (1976), 64.

26. Thompson, K. C., *Analyst*, **100** (1975), 307.

27. Wez, B. and Melcher, M., *Analyst*, **108** (1983), 213.

28. Ebdon, L., Ward, R. W. and Leathard, D. A., *Analyst*, **107** (1982), 129.

29. Ebdon, L. and Ward, R. W., *International Labmate*, **7** (1982).

30. Renoe, B. W., *Journal of Automatic Chemistry*, **4** (1982), 61.

31. Stockwell, P. B., In *Topics in Automatic Chemical Analysis* Volume 1 (E. Horwood, Chichester, UK, 1979).

32. Dennis, A. L. and Porter, D. G., *Journal of Automatic Chemistry*, **2** (1980), 134.

33. de Galan, L. Private communication.

